# The Change of Public Individual Prevention Practice and Psychological Effect From the Early Outbreak Stage to the Controlled Stage of COVID-19 in China in 2020: Two Cross-Sectional Studies

**DOI:** 10.3389/fpsyg.2021.658571

**Published:** 2021-06-16

**Authors:** Bingfeng Han, Hanyu Liu, Tianshuo Zhao, Bei Liu, Hui Zheng, Yongmei Wan, Fuqiang Cui

**Affiliations:** ^1^Department of Epidemiology and Biostatistics, School of Public Health, Peking University, Beijing, China; ^2^Department of Laboratorial Science and Technology, School of Public Health, Peking University, Beijing, China; ^3^National Immunization Program, Chinese Center for Disease Control and Prevention, Beijing, China

**Keywords:** COVID-19, prevention practice, psychology, anxiety, change

## Abstract

**Background:**

COVID-19 broke out in China and spread rapidly in January and February 2020. Following the prevention and control measures of the Chinese government, the outbreak was gradually brought under control after March. The changes in people’s attention to the epidemic, individual prevention practice and psychological effect from the early outbreak stage to the under controlled stage need to be evaluated.

**Methods:**

Two cross-sectional, population-based online surveys were conducted from January 28 to February 1, 2020 and from February1 to March 18, 2020. Socio-demographic information and individual protective practice were collected and the State-Trait Anxiety Inventory (STAI) was used for measuring anxiety. The range of STAI score was 5–25, and the higher the score, the more anxious it was. The respondents of the two surveys were matched on a one-to-one basis according to their province, gender, age, education, and marriage. Wilcoxon signed ranks test and Mann-Whitney *U* test were used to compare STAI score changes in two stages and in different demographic characteristics.

**Results:**

We included 9,764 individuals in the first survey and 1,669 in the second survey, covering 30 provincial administrative regions in Mainland China. COVID-19 has affected almost every aspect of people’s normal life, especially lifestyle. The proportion of people who paid attention to it every day had dropped from 97.6 to 88.9%. We identified that vast majority people wore masks when they went out. The proportion has declined from 96.5 to 92.4% for hand hygiene and from 98.4 to 95.3% for not attending parties. People’s anxiety (STAI score) across the country has decreased from a median of 19 in the early outbreak stage to a median of 12, including people with all demographic characteristics, but some have increased in 16 provinces.

**Conclusion:**

People’s attention to information about the epidemic has declined slightly, but a high proportion of people maintained good practices such as wearing masks, hand hygiene, and not attending parties. People’s anxiety had generally declined from the early outbreak stage to the under controlled stage, but it was still at a high level.

## Introduction

In December 2019, a cluster of patients with pneumonia of unknown cause (named COVID-19 on February 12, 2020; World Health Organization (WHO), 2020b) linked to a seafood wholesale market was identified in Wuhan, China (Zhu N. et al., 2020). During the first 2 months of the first outbreak, COVID-19 spread rapidly throughout China and caused varying degrees of illness (Guan et al., 2020), and SARS-CoV-2 was laboratory confirmed as the cause of the outbreak (Coronaviridae Study Group of the International Committee on Taxonomy of Viruses, 2020). On January 20, 2020, COVID-19 was recognized as a Class B infectious disease by National Health Commission, and was treated as a Class A infectious disease for prevention and control (National Health Commission, 2020a). As of January 28, 2020 (when the first survey started), COVID-19 infection caused 5,974 cases in Mainland China and the number was growing dramatically (National Health Commission, 2020b). And 80,026 cases have been reported in Mainland China as of March 1, 2020 (when the second survey started) (National Health Commission, 2020c). At that time, the epidemic was basically under control, and the number of new cases per day showed a downward trend. As of March 16, the epicenters of Wuhan and Hubei began to lift restrictions (Leung et al., 2020). COVID-19 has caused a global pandemic, more than 14 million cases and more than 603,000 deaths have been reported in 216 countries and territories by July 20, 2020 (World Health Organization (WHO), 2020a) (see Supplementary Appendix 1 for detailed timeline).

National Health Commission had released six versions of the new coronavirus pneumonia prevention and control protocol (National Health Commission, 2020e). People who resided in China started to take measures to protect themselves against COVID-19, such as staying at home as far as possible, limiting social contacts, hand hygiene, wearing protective masks when they needed to move in public (Chen et al., 2020). As a result of these policies and public information and education campaigns, the effective reproduction number fluctuated above 3.0 in Wuhan before January 26, 2020, decreased to below 1.0 after February 6, 2020, and decreased further to less than 0.3 after March 1, 2020 (Pan et al., 2020), which meant that there would be a decline in the number of cases.

Managing mental health and people’s emotions is fundamental to the psychological aspects for the public health emergency (Mukhtar, 2020). During the early outbreak stage of the COVID-19, more than half of the population developed anxiety (Wang C. et al., 2020), although psychological assistance hotlines have been set up in all cities, providing free 24-h service (National Health Commission, 2020d). In March, the prevention and control of the outbreak improved actively in China, and the psychological status of different people also changed. Therefore, on March 18, the National Health Commission issued a work plan for psychological counseling of COVID-19 (National Health Commission, 2020f).

Understanding the public practice of prevention and psychological status at different stages of the epidemic can improve effectiveness of health risk communications and analyzing their demographic differences can help avoid unequal protection across society. Therefore, we conducted two online surveys to measure the individual protective practice and anxiety in the early outbreak stage and controlled stage of COVID-19 in China, providing references for reassuring citizens and outbreak control.

## Materials and Methods

### Study Population

Two cross-sectional, population-based online surveys were conducted, the first survey was from January 28 to February 1, 2020, and the second survey was from March 1–18, 2020. They were open online questionnaires for the people (1) aged 18 years or above, (2) residing in China, (3) willing to respond, (4) able to complete the questionnaire by mobile phone or computer. The questionnaire answered by the participants would be excluded if (1) the answering time was less than 150 s (because we did not think it was possible to finish in less than 150 s if they answer seriously), (2) two questions for questionnaire quality control were answered incorrectly. We used PASS (Power Analysis and Sample Size, Version: 15.0.5, NCSS Statistical Software, United States) to calculate the necessary sample size on the basis of an expected minimal change of 5% in people’s attention to the epidemic, individual prevention practice and psychological effect from the early outbreak stage to the under controlled stage with α:0.05 and β:0.20. In this study, 1,047 participants at most were required. Considering a possible dropout rate of 20%, at least 1,309 participants in total (see Supplementary Appendix 2).

### Online Questionnaire

We designed a structured Chinese questionnaire and collected data on Wenjuanxing, an online platform providing functions equivalent to Amazon Mechanical Turk. The two independent online questionnaires were sent from the same way (WeChat and MicroBlog), and anyone who sees the questionnaire on the Internet and meets the inclusion criteria could fill in it. After a large number of questionnaires were collected, some samples were excluded according to the exclusion criteria. The questionnaires used in the two surveys were similar, mainly including the following information: (1) the socio-demographic information of the respondents; (2) how often and how people pay attention to information about COVID-19; (3) recommended practices during the outbreak, including wearing masks, personal hygiene practices, not attending parties and proper diet; (4) anxiety toward COVID-19: the five questions short form of the State-Trait Anxiety Inventory (STAI) was used for measuring anxiety. A five points Likert-type scale was used to ascertain the degree of anxiety for five questions (from 1 to 5, 1 = never, 2 = little, 3 = sometimes, 4 = often, 5 = always). We measured “anxiety scores” ranging from 5 to 25. The higher the score, the more anxious it was. Similar STAI has been used many times to evaluate the anxiety of different Chinese people (Han et al., 2020; Tong et al., 2020; Zhu X. et al., 2020). The questionnaire consisted of 25 questions and can be completed in 3–5 min (see Supplementary Appendix 3).

### Data Management and Statistical Analysis

We used SPSS (version 20.0, IBM, New York, United States) and STATA (version 15.1, StataCorp LLC, College Station, Texas, United States) for data cleaning and statistical analysis. Categorical variables were expressed as absolute and relative frequencies in different groups.

The social demographic characteristics (gander, age, education, marriage, and occupation) of the effective respondents in the two surveys will be compared. Age was counted as a categorical variable at 10 years intervals. The number and proportion of each category of these social demographic characteristics were calculated. The component ratio was used to describe the frequency and channel when people obtained information. Direct standardized questionnaire was used to measure practices scores and anxiety scores on different ages of population to improve comparability among provinces. The spatial data analyses of anxiety scores were conducted using Microsoft Excel 2016 (Redmond, Washington, United States). McNemar test was used to compare the individual prevention practice from early outbreak stage to under controlled stage of COVID-19. We matched the respondents of the two surveys on a one-to-one basis according to their province, gender, age, education, and marriage. Wilcoxon signed ranks test was used to compare STAI score changes in two stages. Mann-Whitney *U* test was used to explore the anxiety changes in different demographic characteristics. The significance level was considered when *P*-values were less than 0.05.

### Ethical Approval

This study was approved as ethical exemption by the Peking University Health Science Center Ethics Committee (IRB00001052). All subjects participated agreed to participate in the surveys through oral informed consent, and the information in the database was completely de-identified.

## Results

### Study Participants and Characteristics

Ten thousand nine hundred sixty-six individuals participated in the online survey in the early outbreak stage. Among these, 1,202 were excluded due to answering without serious consideration or incomplete questionnaire, and yielded a rate of completeness was 89.0% (9,764/10,966). One thousand nine hundred thirty-eight individuals participated in the online survey in the under controlled stage. Among these, 269 were excluded due to out of age range or incomplete questionnaire, and the rate of completeness was 86.1% (1,669/1,938). The total rate of completeness was 88.6% (11,433/12,904) (see Supplementary Appendix 4).

The participants covered 30 provincial administrative regions in Mainland China (except Tibet). Four thousand forty-five (35.4%) was male; average age was 38.2 ± 12.0 years old; 10,871 (95.1%) were with senior high school education or above; and 7,880 (68.9%) were unmarried (Table 1).

**TABLE 1:** The characteristic of valid participants in online survey when COVID-19 was early and under control in China, 2020.

	Early outbreak stage		Under controlled stage		Total	
	Number	%	Number	%	Number	%
**Gender**						
Male	3,278	33.6	767	46.0	4,045	35.4
Female	6,486	66.4	902	54.0	7,388	64.6
**Age**						
<30	2,725	27.9	359	21.5	3,084	27.0
30–39	2,858	29.3	486	29.1	3,344	29.2
40–49	2,382	24.4	424	25.4	2,806	24.5
≥50	1,799	18.4	400	24.0	2,199	19.2
**Education**						
Junior high school and below	483	4.9	79	4.7	562	4.9
Senior high school	1,315	13.5	164	9.8	1,479	12.9
Bachelor’s degree	5,549	56.8	997	59.7	6,546	57.3
Master’s degree or above	2,417	24.8	429	25.7	2,846	24.9
**Marriage**						
Married	2,789	28.6	362	21.7	3,151	27.6
Unmarried	6,618	67.8	1,262	75.6	7,880	68.9
Divorced	260	2.7	34	2.0	294	2.6
Other	97	1.0	11	0.7	108	0.9
**Occupation**						
Medical professional	299	3.1	50	3.0	349	3.1
Labors	661	6.8	130	7.8	791	6.9
Teachers and researchers	2,662	27.3	568	34.0	3,230	28.3
C&S personnel	397	4.1	84	5.0	481	4.2
Students	221	2.3	45	2.7	266	2.3
Other^#^	5,524	56.6	792	47.5	6,316	55.2
**Total**	9,764	100.0	1,669	100.0	11,433	100.0

*C&S, Commercial and service personnel. ^#^Including farmer, civil servant, self-employed, driver, retired people, unemployed, etc.*

### Impact of COVID-19 on People Who Resided in China

**FIGURE 1:**
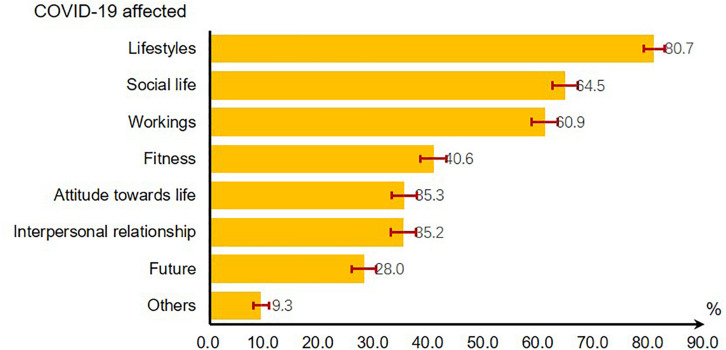
The impact of COVID-19 on people’s life in China.

As of mid-March, COVID-19 has affected almost every aspect of people’s normal life, especially lifestyle. 80.7% reported that their lifestyle was affected by the outbreak. More than 60% of people reported harm to their social life (64.5%) and workings (60.9%) (Figure 1).

### Changes in People’s Attention and Channel Preference for COVID-19

**FIGURE 2:**
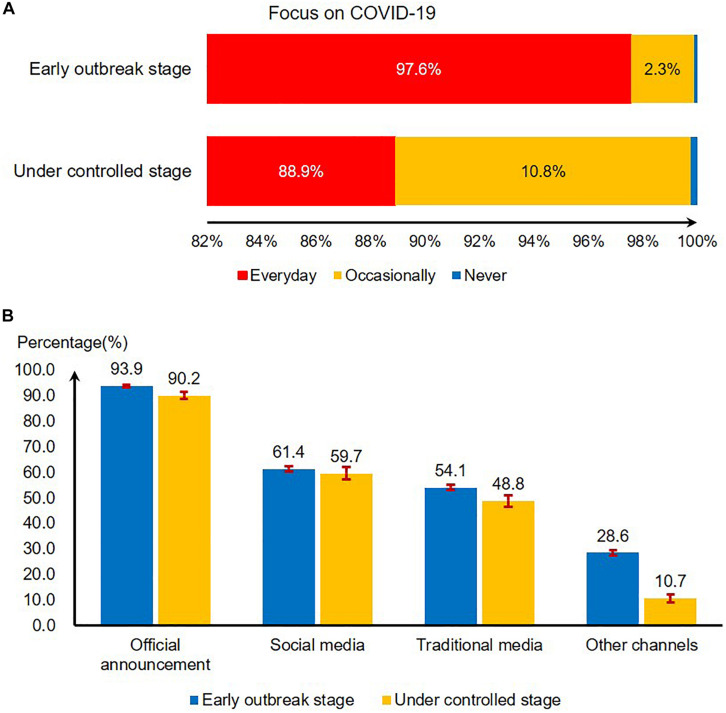
The changes of people’s attention and channel preference for COVID-19 outbreak information when COVID-19 was early and under control in China, 2020. **(A)** People’s attention to COVID-19 when COVID-19 was early and under control in China, 2020. **(B)** Channel preference for COVID-19 outbreak information reporting and seeking when COVID-19 was early and under control in China, 2020.

In the early outbreak stage, 97.6% of people (9,525/9,764) paid daily attention to COVID-19 information. But in the under controlled stage, the proportion of people who paid daily attention to it had dropped to 88.9% (1,484/1,669), and the proportion of people who occasionally pay attention to it had risen from 2.3 to 10.8% (Figure 2A). All the changes were statistically significant (χ^2^ = 274.4, *P* < 0.01).

The proportion of people choosing various channels to obtain information has declined. More than 90% of the respondents obtained epidemic information through official announcement (93.9% in the early outbreak stage and 90.2% in the under controlled stage), followed by social media (61.4% in the early outbreak stage and 59.7% in the under controlled stage) and traditional media (54.1% in the early outbreak stage and 48.8% in the under controlled stage) (Figure 2B).

### Individual Prevention Practice From Early Outbreak Stage to Under Controlled Stage

Almost all people wore masks when they went out (97.9% in the early outbreak stage and 98.9% in the under controlled stage), and there is no statistically significant decrease (χ^2^ = 3.84, *P* = 0.05). The proportion of hand hygiene and not attending parties recommended by the government has declined from the early outbreak stage to the under controlled stage (from 96.5 to 92.4% for hand hygiene, from 98.4 to 95.3% for not attending parties), and the decrease is statistically significant (χ^2^ = 26.41, *P* < 0.01, χ^2^ = 24.01, *P* < 0.01) (Table 2).

**TABLE 2:** Individual prevention practice from early outbreak stage to under controlled stage of COVID-19 in China, 2020.

	Number	Do it (%)		χ^2^*	*P*
		Early outbreak stage	Under controlled stage		
Wearing masks	1,605	1,572 (97.9)	1,587 (98.9)	3.84	0.05
Hand hygiene	1,605	1,549 (96.5)	1,483 (92.4)	26.41	<0.01
Not attending parties	1,605	1,580 (98.4)	1,530 (95.3)	24.01	<0.01
Proper diet	1,605	1,126 (70.2)	1,102 (68.7)	0.85	0.36

*McNemar test.

### Anxiety Changes From Early Outbreak Stage to Under Controlled Stage

People’s anxiety (STAI score) across the country has decreased from a median of 19 in the early outbreak stage to a median of 12 in the under controlled stage, and the decrease is statistically significant (*Z* = 30.5, *P* < 0.01). All items included in the anxiety score have a statistically significant decrease (Table 3A).

**TABLE 3A:** The level of anxiety of participants from early outbreak stage to under controlled stage of COVID-19 in China, 2020.

	Number	Median (P25, P75)		Z*	*P*
		Early outbreak stage	Under controlled stage		
**Anxiety score**	**1,605**	**19 (15, 23)**	**12 (9, 15)**	**-30.47**	**<0.01**
Nervous	1,605	3 (3, 4)	3 (2, 3)	−21.23	<0.01
Fearing	1,605	4 (3, 5)	2 (1.5, 3)	−26.52	<0.01
Angry	1,605	4 (3, 5)	2 (1, 3)	−29.11	<0.01
Pessimistic	1,605	4 (3, 5)	2 (1, 3)	−30.29	<0.01
Tired	1,605	4 (3, 5)	3 (2, 3)	−25.84	<0.01

*Wilcoxon signed ranks test.

**FIGURE 3:**
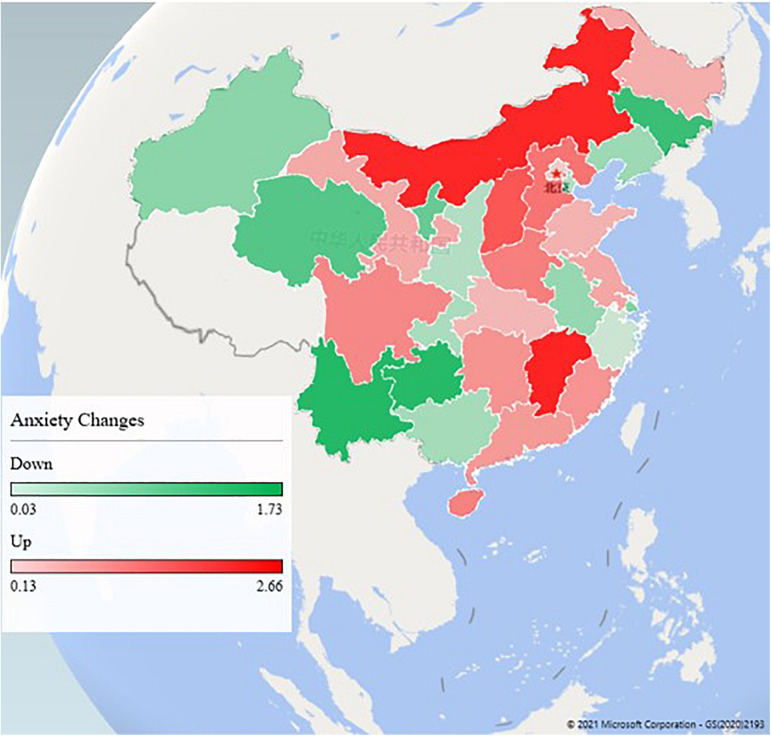
The change of anxiety from early outbreak stage to under controlled stage in different provinces in China.

Based on the standardized anxiety scores, we found that people’s anxiety scores increased in 16 provinces, and decreased in the remaining 14 provinces. People’s anxiety increased most in Jiangxi Province (increased by 2.66 units), followed by Inner Mongolia (increased by 2.60 units). The province with the greatest reduction in people’s anxiety was Yunnan Province (reduced by 1.73 units), followed by Guizhou Province (reduced by 1.70 units) (Figure 3).

At the beginning of the outbreak, men, older people, and those with junior high school education and below were more anxious. Decreased anxiety scores in male (average drop of 7.72 units) was more than that in female (average drop of 6.34 units), and the difference is statistically significant (*Z* = −4.63, *P* < 0.01). Decreased anxiety score in people over age of 50 (average drop of 9.08 units) was significantly more than that in people under age of 30 (average drop of 6.20 units) (*Z* = −6.80, *P* < 0.01). Decreased anxiety scores in people with senior high school or above (average drop of 8.38, 6.93, 6.00 units for senior high school, bachelor’s degree, and master’s degree or above, respectively) were significantly less than that in people with junior high school and below (average drop of 10.16 units). Unmarried people had dropped more anxiety than married people (*Z* = −3.08, *P* < 0.01). Medical professionals and labors have higher anxiety than other occupations in the early outbreak stage. In the under controlled stage, their anxiety had dropped significantly and was not different from that in people with other occupations (Table 3B).

**TABLE 3B:** Anxiety changes in different demographic characteristics from early outbreak stage to under controlled stage of COVID-19 in China, 2020.

	Number	Median (P25, P75)		Average reduction	Z*	*P*
		Early outbreak stage	Under controlled stage			
**Gender**						
Male^*Ref*^	730	20 (16, 24)	12 (9, 15)	7.72		
Female	875	18 (15, 22)	12 (9, 15)	6.34	−4.63	<0.01
**Age**						
<30^*Ref*^	347	19 (15, 22)	13 (9, 15)	6.20		
30–39	477	18 (15, 22)	13 (10, 15)	5.29	−2.24	0.03
40–49	405	20 (16, 23)	12 (9, 15)	7.64	−3.51	<0.01
≥50	376	20 (17, 23)	11 (7, 14)	9.08	−6.80	<0.01
**Education**						
Junior high school and below^*Ref*^	68	21.5 (17, 25)	11 (8, 14)	10.16		
Senior high school	154	19 (16, 23.25)	11 (7.75, 14)	8.38	−2.07	0.04
Bachelor’s degree	975	19 (15, 22)	12 (9, 15)	6.93	−4.13	<0.01
Master’s degree or above	408	19 (15, 22)	13 (10, 15)	6.00	−4.88	<0.01
**Marriage**						
Married^*Ref*^	353	19 (15, 22)	12 (9, 15)	6.05		
Unmarried	1,228	19 (16, 23)	12 (9, 15)	7.21	−3.08	<0.01
Divorced	20	21 (18, 22.75)	12 (9.25, 15.75)	7.45	−1.26	0.21
Other	4	17 (16, 20.25)	6.5 (5, 13.25)	9.50	−1.29	0.20
**Occupation**						
Medical professional^*Ref*^	46	21 (15, 23)	13 (8, 17)	6.59		
Labors	117	21 (17, 24)	12 (9, 15)	8.29	−1.49	0.14
Teachers and researchers	552	19 (15, 23)	12 (9, 15)	6.43	−0.10	0.92
C&S personnel	83	20 (17, 23)	11 (8, 15)	8.27	−1.61	0.11
Students	45	17 (15, 21)	13 (9.5, 15.5)	5.69	−0.64	0.52
Other^#^	762	19 (15, 22)	12 (8, 15)	7.11	−0.51	0.61
**Total**	1,605	19 (15, 23)	12 (9, 15)	6.97		

C&S, commercial and service personnel; Ref, reference group. *Mann-Whitney U test. ^#^Including farmer, civil servant, self-employed, driver, retired people, unemployed, etc.

## Discussion

Our study demonstrated that COVID-19 outbreak has a great negative impact on people who resided in China, especially lifestyle, social life, and workings. Some people had reduced their attention to the epidemic information in the under controlled stage, but people were still very concerned about the outbreak in China and abroad. People have performed well on the individual prevention practice recommended by the government, although their anxiety has decreased significantly from the early outbreak stage to the under controlled stage.

A stringent confinement of people in high risk areas seems to have a potential to slow down the spread of COVID-19 (Lau et al., 2020), but people’s lifestyles and social styles have also changed. They attempted avoiding unnecessary face-to-face communication. Physical contact was transferred to virtual meeting. The finding suggests that high proportion of people followed the government recommendations and kept the safe physical distance during the outbreak. By April 2020, China has basically blocked the local transmission of COVID-19. On May 7, 2020, the State Council issued guidance on the normalized prevention and control of COVID-19 (State Council, 2020). On the one hand, it is necessary to prevent imported cases from abroad, on the other hand, it should allow people to move reasonably and promote the resumption of working and production in an all-round way.

This study showed that respondents’ attention to COVID-19 had declined from the early outbreak stage to the under controlled stage. According to the Baidu Index of people who resided in China searching for “pneumonia,” there was a peak in the search index from late January to mid-February, and then it returned to the average (Baidu Index, 2020). People’s attention to the risk of infection was more rational, and people’s sense of security has been improved.

People attach great importance to individual prevention practice. This study found that people maintain a good practice of wearing masks when going out from the early outbreak stage to the under controlled stage. Almost all people still insist on hand hygiene and not attending parties recommended by the government, although the proportion has declined slightly. Other surveys in China also show similarly high proportions (Liu et al., 2020). Various regions in China have successively activated first-level public health emergency response, and released timely information on prevention and control of the outbreak. People who resided in China responded to the requirement of “wearing masks, hand hygiene, and not attending parties” and actively fought the epidemic.

This outbreak was also leading to additional psychological problems such as stress, anxiety, depressive symptoms, insomnia, denial, anger, and fear globally (Torales et al., 2020). A survey conducted during the H1N1 influenza pandemic in 2009 indicated the importance of precise and clear information about control measures for reducing anxiety (Taha et al., 2014). This study found that people who resided in China were more anxious at the beginning of the outbreak. Another study of the same period showed that Wuhan residents’ psychological status and sleep quality were relatively poorer than they were before the COVID-19 epidemic (Fu et al., 2020). Similar results were found not only in China, but also in India (Varalakshmi and Swetha, 2020) and Italy (Moccia et al., 2020). This study found that people over age of 50 were more anxious. COVID-19 has proven to be particularly deadly to older adults (Nanda et al., 2020; Nikolich-Zugich et al., 2020), which accumulates stress and fear among them (Meng et al., 2020). This study also found that people with junior high school education and below were more anxious, which was similar to the results of another study in China (Lei et al., 2020). Recent works have shown that the heightened perceived risk of financial loss due to COVID-19 was highest among those with the lowest education and income (Simone and Natalie, 2020), which may lead to anxiety among those people. However, there are some articles that report different results on the relationship between age and education level and anxiety (Ahmed et al., 2020; Islam et al., 2020). Medical professionals and labors have higher anxiety than other occupations in the early outbreak stage maybe due to high knowledge among medical professionals and they valued high risk for the outbreak than other occupations, who should be paid more attention (Dong et al., 2020; Que et al., 2020; Teng et al., 2020; Xiao et al., 2020; Zhu Z. et al., 2020). This study found that unmarried people had dropped more anxiety than married people, which was similar to another study in China (Wang H. et al., 2020). The possible reason was that unmarried people need to bear more economic and living burden brought by the epidemic. When the epidemic was basically under control, people’s anxiety (STAI score) had generally declined, but it was still at a high level. We found that people’s anxiety was still on the rise in Jiangxi Province and Inner Mongolia, which may have a negative impact on normal life and work of people in these provinces. In particular, there are long borders in Inner Mongolia, leading to a high risk of imported cases from abroad, which may cause public anxiety. These changes needed to arouse the awareness of the local government.

According to an interesting experience from Denmark, combining the professional angles of psychology and infection prevention proved fruitful (Olesen et al., 2020). COVID-19 outbreak had attracted widespread public attention in China. Most people obtained information on the outbreak and individual protection practice through online official media and social media, which makes it possible for the public to psychosocial intervention based on the internet (Yang et al., 2020), including viewing heroic acts, speeches from experts, and knowledge of the disease and prevention (Chao et al., 2020).

There are some limitations to our study. First, online survey induced a selection biased. The respondents are mainly those living in urban area and with high school education or above, which may overestimate the knowledge of the outbreak and protection. Second, although we have carried out quality control, there may be errors in the information because the online questionnaire cannot be modified after filling in. Third, it was hard to get a comprehensive understanding of anxiety from the five questions short form of STAI, but it can also reflect some characteristics and provide reference. Finally, the difference in sample size between the two cross-sectional studies was large, and two cross-sectional surveys to describe people’s individual protection practice and psychological effects are not comprehensive enough, so more similar surveys are still needed for longitudinal study.

In summary, the public’s lifestyle has been impacted by the epidemic, but people’s attention to information about the epidemic has declined slightly from early stage of outbreak to under controlled stage. A high proportion of people maintained good practices such as wearing masks, hand hygiene, and not attending parties. People’s anxiety had generally declined from the early outbreak stage to the under controlled stage, but it was still at a high level. Our findings suggest that online psychological counseling and health education is needed to reduce psychological anxiety of people.

## Data Availability Statement

The raw data supporting the conclusions of this article will be made available by the authors, without undue reservation.

## Ethics Statement

Ethical review and approval was not required because the research was deemed exempt by the Peking University Health Science Center Ethics Committee (IRB00001052). Written informed consent for participation was not required for this study in accordance with the national legislation and the institutional requirements.

## Author Contributions

BH, HL, and TZ wrote the first draft. BH, HL, TZ, BL, and YW completed two surveys. HZ and FC made major revisions to the logic of this article. BH, HL, TZ, HZ, and FC participated in the discussion of the manuscript. All authors approved the final version of the manuscript for submission.

## Conflict of Interest

The authors declare that the research was conducted in the absence of any commercial or financial relationships that could be construed as a potential conflict of interest. The reviewer FW declared a shared affiliation, with several of the authors HZ and YW to the handling Editor at the time of the review.

## References

[B1] Ahmed M. Z. Ahmed O. Aibao Z. Hanbin S. Siyu L. Ahmad A. (2020). Epidemic of COVID-19 in China and associated psychological problems. *Asian J. Psychiatr.* 51:102092. 10.1016/j.ajp.2020.102092 32315963 PMC7194662

[B2] Baidu Index (2020). *Research on the Trend of “Pneumonia” Search Index. Last Modified 2020-07-27.* Available online at: https://index.baidu.com/v2/main/index.html#/trend/%E8%82%BA%E7%82%8E?words=%E8%82%BA%E7%82%8E,%E6%96%B0%E5%86%A0 (accessed July 28, 2020).

[B3] Chao M. Dini X. Tour L. Haibo Y. Brian J. H. (2020). Media use and acute psychological outcomes during COVID-19 outbreak in China. *J. Anxiety Disord.* 74 102248–102248. 10.1016/j.janxdis.2020.102248 32505918 PMC7255752

[B4] Chen S. M. Yang J. T. Yang W. Z. Wang C. Barnighausen T. (2020). COVID-19 control in China during mass population movements at New Year. *Lancet* 395 764–766. 10.1016/S0140-6736(20)30421-9 32105609 PMC7159085

[B5] Coronaviridae Study Group of the International Committee on Taxonomy of Viruses (2020). The species Severe acute respiratory syndrome-related coronavirus: classifying 2019-nCoV and naming it SARS-CoV-2. *Nat. Microbiol.* 5 536–544. 10.1038/s41564-020-0695-z 32123347 PMC7095448

[B6] Dong Z. Q. Ma J. Hao Y. N. Shen X. L. Liu F. Gao Y. (2020). The social psychological impact of the COVID-19 pandemic on medical staff in China: a cross-sectional study. *Eur. Psychiatry* 63:e65. 10.1192/j.eurpsy.2020.59 32476633 PMC7343668

[B7] Fu W. Wang C. Zou L. Guo Y. Lu Z. Yan S. (2020). Psychological health, sleep quality, and coping styles to stress facing the COVID-19 in Wuhan, China. *Transl. Psychiatry* 10:225. 10.1038/s41398-020-00913-3 32647160 PMC7347261

[B8] Guan W. J. Ni Z. Y. Hu Y. Liang W. H. Ou C. Q. He J. X. (2020). Clinical characteristics of coronavirus disease 2019 in China. *N. Engl. J. Med.* 382 1708–1720. 10.1056/NEJMoa2002032 32109013 PMC7092819

[B9] Han Y. Fan J. Wang X. Xia J. Liu X. Zhou H. (2020). Factor structure and gender invariance of chinese version state-trait anxiety inventory (Form Y) in university students. *Front. Psychol.* 11:2228. 10.3389/fpsyg.2020.02228 33132946 PMC7578736

[B10] Islam M. S. Most Z. F. Marc N. P. (2020). Panic and generalized anxiety during the COVID-19 pandemic among Bangladeshi people: an online pilot survey early in the outbreak. *J. Affect. Disord.* 276 30–37. 10.1016/j.jad.2020.06.049 32697713 PMC7362838

[B11] Lau H. Khosrawipour V. Kocbach P. Mikolajczyk A. Schubert J. Bania J. (2020). The positive impact of lockdown in Wuhan on containing the COVID-19 outbreak in China. *J. Travel Med.* 27:taaa037. 10.1093/jtm/taaa037 32181488 PMC7184469

[B12] Lei L. Huang X. Zhang S. Yang J. Yang L. Xu M. (2020). Comparison of prevalence and associated factors of anxiety and depression among people affected by versus people unaffected by quarantine during the COVID-19 Epidemic in Southwestern China. *Med. Sci. Moni.t* 26:e924609. 10.12659/MSM.924609 32335579 PMC7199435

[B13] Leung K. Wu J. T. Liu D. Leung G. M. (2020). First-wave COVID-19 transmissibility and severity in China outside Hubei after control measures, and second-wave scenario planning: a modelling impact assessment. *Lancet* 395 1382–1393. 10.1016/S0140-6736(20)30746-7 32277878 PMC7195331

[B14] Liu X. Luo W. T. Li Y. Li C. N. Hong Z. S. Chen H. L. (2020). Psychological status and behavior changes of the public during the COVID-19 epidemic in China. *Infect. Dis. Poverty* 9:58. 10.1186/s40249-020-00678-3 32471513 PMC7256340

[B15] Meng H. Yang X. Dai J. Zhang Y. Liu B. Yang H. (2020). Analyze the psychological impact of COVID-19 among the elderly population in China and make corresponding suggestions. *Psychiatr. Res.* 289:112983. 10.1016/j.psychres.2020.112983 PMC7151427 32388175

[B16] Moccia L. Janiri D. Pepe M. Dattoli L. Molinaro M. De Martin V. (2020). Affective temperament, attachment style, and the psychological impact of the COVID-19 outbreak: an early report on the Italian general population. *Brain Behav. Immun.* 87 75–79. 10.1016/j.bbi.2020.04.048 32325098 PMC7169930

[B17] Mukhtar S. (2020). Feminism and gendered impact of COVID-19: perspective of a counselling psychologist. *Gender Work Organ.* 27 827–832. 10.1111/gwao.12482 32837011 PMC7300594

[B18] Nanda A. Nvrk V. Gravenstein S. (2020). COVID-19 in older adults. *Aging Clin. Exp. Res.* 32 1199–1202. 10.1007/s40520-020-01581-5 32390064 PMC7211267

[B19] National Health Commission (2020a). *Announcement of the National Health Commission(2020 No. 1). Last Modified 2020-01-20.* Available online at: http://www.nhc.gov.cn/jkj/s7916/202001/44a3b8245e8049d2837a4f27529cd386.shtml (accessed July 26, 2020).

[B20] National Health Commission (2020b). *The Latest Situation of COVID-19 Outbreak as of January 28. Last Modified 2020-02-29.* Available online at: http://www.nhc.gov.cn/xcs/yqtb/202001/1c259a68d81d40abb939a0781c1fe237.shtml (accessed July 21, 2020).

[B21] National Health Commission. (2020c). *The Latest Situation of COVID-19 Outbreak as of March 1. Last Modified 2020-03-02.* Available online at: http://www.nhc.gov.cn/xcs/yqtb/202003/5819f3e13ff6413ba05fdb45b55b66ba.shtml (accessed July 21, 2020).

[B22] National Health Commission (2020d). *Notice on Establishing a Psychological Assistance Hotline for Outbreak Response. Last Modified 2020-02-02.* Available online at: http://www.nhc.gov.cn/jkj/s3577/202002/8f832e99f446461a87fbdceece1fdb02.shtml (accessed July 21, 2020).

[B23] National Health Commission (2020e). *Prevention and Control of Novel Coronavirus Pneumonia. Last Modified 2020-03-27.* Available online at: http://www.nhc.gov.cn/jkj/s3577/202003/4856d5b0458141fa9f376853224d41d7/files/4132bf035bc242478a6eaf157eb0d979.pdf (accessed July 21, 2020).

[B24] National Health Commission (2020f). *Work Plan for Psychological Counseling of COVID-19. Last Modified 2020-03-18.* Available onlilne at: http://www.nhc.gov.cn/jkj/s3577/202003/0beb22634f8a4a48aecf405c289fc25e.shtml (accessed July 27, 2020).

[B25] Nikolich-Zugich J. Knox K. S. Rios C. T. Natt B. Bhattacharya D. Fain M. J. (2020). SARS-CoV-2 and COVID-19 in older adults: what we may expect regarding pathogenesis, immune responses, and outcomes. *Geroscience* 42 505–514. 10.1007/s11357-020-00186-0 32274617 PMC7145538

[B26] Olesen B. Gyrup H. B. Troelstrup M. W. Marloth T. Molmer M. (2020). Infection prevention partners up with psychology in a Danish Hospital successfully addressing staffs fear during the COVID-19 pandemic. *J. Hosp. Infect.* 105 377–378. 10.1016/j.jhin.2020.04.033 32339619 PMC7194839

[B27] Pan A. Liu L. Wang C. Guo H. Hao X. Wang Q. (2020). Association of public health interventions with the epidemiology of the COVID-19 outbreak in Wuhan, China. *JAMA* 323 1915–1923. 10.1001/jama.2020.6130 32275295 PMC7149375

[B28] Que J. Shi L. Deng J. Liu J. Zhang L. Wu S. (2020). Psychological impact of the COVID-19 pandemic on healthcare workers: a cross-sectional study in China. *Gen. Psychiatr.* 33:e100259. 10.1136/gpsych-2020-100259 32596640 PMC7299004

[B29] Simone S. Natalie T. (2020). *Individuals with Low Incomes, Less Education Report Higher Perceived Financial, Health Threats from COVID-19. Last Modified 2020-04-01.* Available online at: https://healthpolicy.usc.edu/evidence-base/individuals-with-low-incomes-less-education-report-higher-perceived-financial-health-threats-from-covid-19/ (accessed May 25, 2021).

[B30] State Council (2020). *Guidance on the Normalized Prevention and Control of COVID-19. Last Modified 2020-05-07.* Available online at: http://www.gov.cn/zhengce/content/2020-05/08/content_5509896.htm (accessed July 27, 2020).

[B31] Taha S. Matheson K. Cronin T. Anisman H. (2014). Intolerance of uncertainty, appraisals, coping, and anxiety: the case of the 2009 H1N1 pandemic. *Br. J. Health Psychol.* 19 592–605. 10.1111/bjhp.12058 23834735

[B32] Teng Z. Wei Z. Qiu Y. Tan Y. Chen J. Tang H. (2020). Psychological status and fatigue of frontline staff two months after the COVID-19 pandemic outbreak in China: a cross-sectional study. *J. Affect. Disord.* 275 247–252. 10.1016/j.jad.2020.06.032 32734915 PMC7330556

[B33] Tong Q. Y. Liu R. Zhang K. Gao Y. Cui G. W. Shen W. D. (2020). Can acupuncture therapy reduce preoperative anxiety? A systematic review and meta-analysis. *J. Integr. Med.* 19 20–28. 10.1016/j.joim.2020.10.007 33288487

[B34] Torales J. O’Higgins M. Castaldelli-Maia J. M. Ventriglio A. (2020). The outbreak of COVID-19 coronavirus and its impact on global mental health. *Int. J. Soc. Psychiatr.* 66 317–320. 10.1177/0020764020915212 32233719

[B35] Varalakshmi R. Swetha R. (2020). Covid-19 lock down: People psychology due to law enforcement. *Asian J. Psychiatr.* 51 102102–102102. 10.1016/j.ajp.2020.102102 32344332 PMC7162767

[B36] Wang C. Pan R. Wan X. Tan Y. Xu L. Ho C. S. (2020). Immediate psychological responses and associated factors during the initial stage of the 2019 Coronavirus disease (COVID-19) epidemic among the general population in China. *Int. J. Environ. Res. Public Health* 17:1729. 10.3390/ijerph17051729 32155789 PMC7084952

[B37] Wang H. Xia Q. Xiong Z. Li Z. Xiang W. Yuan Y. (2020). The psychological distress and coping styles in the early stages of the 2019 coronavirus disease (COVID-19) epidemic in the general mainland Chinese population: a web-based survey. *PLoS One* 15:e0233410. 10.1371/journal.pone.0233410 32407409 PMC7224553

[B38] World Health Organization (WHO) (2020a). *Coronavirus Disease (COVID-19) Situation Report - 182. Last Modified 2020-07-20.* Available online at: https://www.who.int/docs/default-source/coronaviruse/situation-reports/20200720-covid-19-sitrep-182.pdf?sfvrsn=60aabc5c_2 (accessed July 21, 2020).

[B39] World Health Organization (WHO) (2020b). *WHO Director-General’s Remarks at the Media Briefing on 2019-nCoV on 11 February 2020. Last Modified 2020-02-11.* Available online at: https://www.who.int/dg/speeches/detail/who-director-general-s-remarks-at-the-media-briefing-on-2019-ncov-on-11-february-2020 (accessed February 12, 2020).

[B40] Xiao X. Zhu X. Fu S. Hu Y. Li X. Xiao J. (2020). Psychological impact of healthcare workers in China during COVID-19 pneumonia epidemic: a multi-center cross-sectional survey investigation. *J. Affect. Disord.* 274 405–410. 10.1016/j.jad.2020.05.081 32663970 PMC7236675

[B41] Yang J. Tong J. Meng F. Feng Q. Ma H. Shi C. (2020). Characteristics and challenges of psychological first aid in China during the COVID-19 outbreak. *Brain Behav. Immun.* 87 113–114. 10.1016/j.bbi.2020.04.075 32353519 PMC7185020

[B42] Zhu N. Zhang D. Wang W. Li X. Yang B. Song J. (2020). A novel coronavirus from patients with pneumonia in China, 2019. *N. Engl. J. Med.* 382 727–733. 10.1056/NEJMoa2001017 31978945 PMC7092803

[B43] Zhu X. Lu Q. Yao Y. Xu X. Lu Y. (2020). Intraoperative pain sensation during cataract surgery: why does timing matter? *Curr. Eye Res.* 42:30Z. 10.1080/02713683.2020.1857776 33249933

[B44] Zhu Z. Liu Q. Jiang X. Manandhar U. Luo Z. Zheng X. (2020). The psychological status of people affected by the COVID-19 outbreak in China. *J. Psychiatr. Res.* 129 1–7. 10.1016/j.jpsychires.2020.05.026 32526513 PMC7255091

