# Supplementary material for: The Change of Public Individual Prevention Practice and Psychological Effect From the Early Outbreak Stage to the Controlled Stage of COVID-19 in China in 2020: Two Cross-Sectional Studies
Source: Front Psychol. 2021 Jun 16;12:658571. doi: 10.3389/fpsyg.2021.658571 (PMC8242258; doi:10.3389/fpsyg.2021.658571)
Supplement: Supplementary file 1 [file Data_Sheet_1.docx]

Appendix 1 Timeline of COVID-19 events

**References:**

[1] WHO Timeline - COVID-19 [homepage on the Internet]; [updated 2020-04-27]. Available from: <https://www.who.int/news-room/detail/27-04-2020-who-timeline---covid-19>

[2] Record of COVID-19 epidemic information in China [homepage on the Internet]; [updated 2020-04-06]. Available from: <http://www.gov.cn/xinwen/2020-04/06/content_5499625.htm>
