# Supplementary material for: The Change of Public Individual Prevention Practice and Psychological Effect From the Early Outbreak Stage to the Controlled Stage of COVID-19 in China in 2020: Two Cross-Sectional Studies
Source: Front Psychol. 2021 Jun 16;12:658571. doi: 10.3389/fpsyg.2021.658571 (PMC8242258; doi:10.3389/fpsyg.2021.658571)
Supplement: Supplementary file 2 [file Data_Sheet_2.docx]

**Appendix 2**

**Sample size:**

**Numeric Results for Testing One Proportion using the Exact Test**

Alternative Hypothesis: Two-Sided (H0: P = P0 vs. H1: P ≠ P0)

| Power* | n | Proportion Given H0 | Proportion Given H1 | P1-P0 | Alpha | If R ≤ \| R ≥ |
| --- | --- | --- | --- | --- | --- | --- |
|  |  | P0 | P1 |  |  |  |
| 0.90286 | 62 | 0.5000 | 0.3000 | -0.2000 | 0.0500 | 22\|40 |
| 0.90120 | 113 | 0.5000 | 0.3500 | -0.1500 | 0.0500 | 45\|68 |
| 0.90055 | 259 | 0.5000 | 0.4000 | -0.1000 | 0.0500 | 113\|146 |
| 0.90011 | 1047 | 0.5000 | 0.4500 | -0.0500 | 0.0500 | 491\|556 |

* Power was computed using the normal approximation method.

**Report Definitions**

Power is the probability of rejecting the null hypothesis when it is false. It should be close to one.

n is the size of the sample drawn from the population. To conserve resources, it should be as small as possible.

P0 is the value of the population proportion under the null hypothesis.

P1 is the value of the population proportion under the alternative hypothesis.

P1-P0 is the difference to be detected by the study.

Alpha (significance level) is the probability of rejecting the null hypothesis when it is true. It should be small.

Reject H0 If... gives the critical value(s) for the test.

**References**

Chow, S. C., Shao, J., and Wang, H. 2008. Sample Size Calculations in Clinical Research, Second Edition. Chapman & Hall/CRC. Boca Raton, Florida.

Fleiss, J. L., Levin, B., and Paik, M.C. 2003. Statistical Methods for Rates and Proportions. Third Edition. John Wiley & Sons. New York.

Lachin, John M. 2000. Biostatistical Methods. John Wiley & Sons. New York.

Machin, D., Campbell, M., Fayers, P., and Pinol, A. 1997. Sample Size Tables for Clinical Studies, 2nd Edition. Blackwell Science. Malden, Mass.

Ryan, Thomas P. 2013. Sample Size Determination and Power. John Wiley & Sons. Hoboken, New Jersey.

Zar, Jerrold H. 2010. Biostatistical Analysis (Fifth Edition). Prentice-Hall. Englewood Cliffs, New Jersey.

**Summary Statements**

A sample size of 62 achieves 90.286% power to detect a difference (P1-P0) of -0.2000 using a two-sided exact test with a significance level (alpha) of 0.0500. These results assume that the population proportion under the null hypothesis (P0) is 0.5000.

**Dropout-Inflated Sample Size**

| Dropout Rate | Sample Size | Dropout-Inflated Enrollment Sample Size | Expected Number of Dropouts |
| --- | --- | --- | --- |
|  | n | n' | D |
| 20% | 62 | 78 | 16 |
| 20% | 113 | 142 | 29 |
| 20% | 259 | 324 | 65 |
| 20% | 1047 | 1309 | 262 |

**Definitions**

Dropout Rate (DR) is the percentage of subjects (or items) that are expected to be lost at random during the course of the study and for whom no response data will be collected (i.e. will be treated as "missing").

n is the evaluable sample size at which power is computed. If n subjects are evaluated out of the n' subjects that are enrolled in the study, the design will achieve the stated power.

n' is the total number of subjects that should be enrolled in the study in order to end up with n evaluable subjects, based on the assumed dropout rate. After solving for n, n' is calculated by inflating n using the formula n' = n / (1 - DR), with n' always rounded up. (See Julious, S.A. (2010) pages 52-53, or Chow, S.C., Shao, J., and Wang, H. (2008) pages 39-40.)

D is the expected number of dropouts. D = n' - n.

**Chart Section**

**Design Tab**

| Solve For | Sample Size |
| --- | --- |
| Power Calculation Method: | Normal Approximation |
| Alternative Hypothesis: | Two-Sided |
| Test Type: | Exact Test |
| N (Population Size): | Infinite |
| Power: | 0.90 |
| Alpha: | 0.05 |
| Input Type: | Proportions |
| P0 (Null Proportion): | 0.50 |
| P1 (Alternative Proportion): | 0.3 0.35 0.4 0.45 |
