# Supplementary material for: The Change of Public Individual Prevention Practice and Psychological Effect From the Early Outbreak Stage to the Controlled Stage of COVID-19 in China in 2020: Two Cross-Sectional Studies
Source: Front Psychol. 2021 Jun 16;12:658571. doi: 10.3389/fpsyg.2021.658571 (PMC8242258; doi:10.3389/fpsyg.2021.658571)
Supplement: Supplementary file 3 [file Data_Sheet_3.docx]

**Appendix 3**

**Questionnaire on prevention practice and psychological effect of COVID-19 in 2020**

**Informed Consent**

There is an online survey, designed by School of public health, Peking University, aiming to understand the public knowledge, practice of prevention, and psychological status of Corona Virus Disease 2019(COVID-19). This survey does not involve any privacy or sensitive issues, and the results of the it will be helpful to China's epidemic management.

**Part A. Relevant information acquisition of COVID-19**

A1. How often are you concerned about the outbreak?

a. Every day

b. Occasionally

c. Don't care

A2. Where do you get information about the outbreak?

[You can choose more than one]

a. Government and professional organizations (including official websites, Weibo and WeChat)

b. News media

c. Search engines (Baidu, Google, etc.)

d. Personal social media

e. TV

f. Radio

g. Community brochure/brochure/column, etc

h. Other mass media (newspapers/magazines)

i. Other (please indicate)

A3. Do you trust the unofficial release of information online? (unofficial: like the grapevine from friends)

a. Completely trust

b. Some trust

c. General trust

d. Some distrust

e. Thoroughly distrustful

A4. What does COVID-19 affect you?

[You can choose more than one]

a. Lifestyles

b. Interpersonal relationship

c. Social life

d. Attitude towards life

e. Fitness

f. Workings

g. Future

h. Other

**Part B. Anxiety about COVID-19**

B1. How did you feel nervous or anxious in the latest week?

a. Frequently

b. Often

c. Generally

d. Less

e. Hardly ever

B2. How did you worry about getting sick in the latest week?

a. Frequently

b. Often

c. Generally

d. Less

e. Hardly ever

B3. How were you prone to anger in the latest week?

a. Frequently

b. Often

c. Generally

d. Less

e. Hardly ever

B4. How were you pessimistic in the latest week?

a. Frequently

b. Often

c. Generally

d. Less

e. Hardly ever

B5. How did you feel tired in the latest week?

a. Frequently

b. Often

c. Generally

d. Less

e. Hardly ever

**Part C. Individual Protection**

C1. What kind of mask do you think can effectively prevent 2019-nCoV infection?

[You can choose more than one]

a. Surgical mask

b. Medical mask (N95 and above)

c. Gauze mask

e. Activated charcoal mask

f. None of them/I don't know

C2. In which environment would you wear a mask?

[You can choose more than one]

a. Parks and roads

b. Supermarkets, malls and other crowded places

c. Small confined Spaces such as elevators

e. Hospital

f. None of them/I don't know

C3. Is there a time limit for the effectiveness of mask wearing?

a. No

b. Yes, 2-4 hours

c. 5-6 hours

d. I don't know

C4. Can hand washing prevent 2019-nCoV infection?

a. Yes

b. No

c. I don't know

C5. What measures have you taken to protect yourself from 2019-nCoV?

[You can choose more than one]

a. Wear a mask when going out

c. Do not use hands when sneezing or coughing

d. Keep hands clean by washing hands correctly and timely

e. Do not go to parties or visits, and try to avoid crowded or enclosed places

f. Eat a healthy balanced diet

**Part D. Sociodemographic Characteristics**

D1. Are you currently living in an urban or rural area?

a. Urban

b. Rural

D2. What is your age?

a. [number]

D3. What was your sex at birth?

a. Male

b. Female

D4. What is your marital status?

a. Never married

b. Engaged or married

c. Separated or divorced

d. Widowed

e. Others

D5. What is your highest level of education?

a. Primary school

b. Junior high school

c. Senior school

d. Some college or a bachelors degree

e. Masters, PhD, or above

D6. What is your main occupation?

a. Labor worker

b. Farmer

c. Teachers and researchers

d. Service/retail

e. Civil servant

f. Health care workers

g. Self-employed

h. Office worker

i. Driver

j. Retired people

k. Student

l. Unemployed

m. Others

D7. How many people are there in your family?

a. 1

b. 2

c. 3

d. 4

e. 5

f. 6

g. 7

h. 8

i. 9

j. 10

D8. Are there any children (under 5) in your family?

a. Yes

b. No

D9. Are there any elderly men or women (over 65) in your family?

a. Yes

b. No

D10. Are there any pregnant women in your family?

a. Yes

b. No

D11. How is your general health?

a. Good

b. About good

c. Fair

e. Bad (under illness)
