# Supplementary material for: The Change of Public Individual Prevention Practice and Psychological Effect From the Early Outbreak Stage to the Controlled Stage of COVID-19 in China in 2020: Two Cross-Sectional Studies
Source: Front Psychol. 2021 Jun 16;12:658571. doi: 10.3389/fpsyg.2021.658571 (PMC8242258; doi:10.3389/fpsyg.2021.658571)
Supplement: Supplementary file 4 [file Data_Sheet_4.docx]

Appendix 4 Inclusion and selection process of respondents
